# Estimation and Analysis of Higher-Order Harmonics in Advanced Integrated Circuits to Implement Noise-Free Future-Generation Micro- and Nanoelectromechanical Systems

**DOI:** 10.3390/mi12050541

**Published:** 2021-05-10

**Authors:** Muhammad Imran Khan, Ahmed S. Alshammari, Badr M. Alshammari, Ahmed A. Alzamil

**Affiliations:** 1 Department of Electrical Engineering, University of Hail, Hail 2440, Saudi Arabia; ahm.alshammari@uoh.edu.sa (A.S.A.); bms.alshammari@uoh.edu.sa (B.M.A.); aa.alzamil@uoh.edu.sa (A.A.A.); 2 Micro-/Nano Electronic System Integration R&D Center (MESIC), University of Science and Technology of China (USTC), Hefei 230027, China

**Keywords:** integrated circuits, flip-flops, latches, micro- and nanosystems, switching harmonics, digital noise

## Abstract

This work deals with the analysis of spectrum generation from advanced integrated circuits in order to better understand how to suppress the generation of high harmonics, especially in a given frequency band, to design and implement noise-free systems. At higher frequencies, the spectral components of signals with sharp edges contain more energy. However, current closed-form expressions have become increasingly unwieldy to compute higher-order harmonics. The study of spectrum generation provides an insight into suppressing higher-order harmonics (10th order and above), especially in a given frequency band. In this work, we discussed the influence of transistor model quality and input signal on estimates of the harmonic contents of switching waveforms. Accurate estimates of harmonic contents are essential in the design of highly integrated micro- and nanoelectromechanical systems. This paper provides a comparative analysis of various flip-flop/latch topologies on different process technologies, i.e., 130 and 65 nm. An FFT plot of the simulated results signifies that the steeper the spectrum roll-off, the lesser the content of higher-order harmonics. Furthermore, the results of the comparison illustrate the improvement in the rise time, fall time, clock-Q delay and spectrum roll-off on the better selection of slow-changing input signals and more accurate transistor models.

## 1. Introduction

Integrated circuit designs are becoming more complex day by day, and the integration of ten billion transistors on the same die with clock frequencies above several gigahertz makes it difficult to implement cost-effective and reliable systems. The development of reliable and cost-effective mixed-signal and RF systems depends on system-on-chip (SOC) and system-in-package (SIP) technologies [1,2,3,4,5]. We need accurate and reliable design tools for the estimation of switching noise coupled from the digital part into analog to implement noise-free integrated circuits for use in future-generation electromechanical systems. Switching noise is of significant importance, as it propagates to the RF section via I/O pins and substrate and can cumulatively reach several hundred millivolts [6,7,8,9,10,11]. Transistor model quality and choice of input signal play a significant role in the exact estimation of switching harmonics. BSIM (Berkeley short-channel insulated-gate field-effect transistor (IGFET) model) transistors models are reliable, accurate and currently considered the industry standard. All simulations presented in this paper are performed on BSIM 3 and BSIM 4 transistor models using Cadence Spectre (V 6.1, Cadence Design Systems, Inc., San Jose, CA, USA) and MATLAB simulators (V 2010a, MathWorks, Inc., Natick, MA, USA).

## 2. Transistor Model Quality

Transistor model parameters influence the spectrum of switching waveforms emitted by digital circuits. With the help of advanced numerical tools, we are able to deduce explicit output-switching waveforms for simple transistor models [10]. Circuit simulations of simple transistor models yield transition edges that are helpful to construct a pulse waveform. The spectrum of the pulse is evaluated through FFT.

Simulations performed with Level 1, Level 2 and Level 3 transistor models show that several discontinuities exist in the form of derivatives of the drain current as a function of terminal voltages. These models did not mitigate the short-channel effects. The spectrum roll-off achieved with these transistor models did not exceed more than 70 dB/decade. However, results with the Maher-mead model display improvement in the spectrum roll-off around 100 dB/decade. Our simulations were performed on de facto industry-standard next-generation device models of the BSIM family. BSIM stands for Berkeley short-channel insulated-gate field-effect transistor model. BSIM models are reliable and provide highly accurate simulation results. Many companies and universities rely on their own MOS transistor models for circuit simulations.

Device threshold voltage is an essential parameter in circuit simulations. Low-threshold devices yield large subthreshold currents. Low-threshold devices turn on softly and produce a steep spectrum roll-off [1]. Due to characteristics, low-threshold voltage devices are good for use in complex integrated circuits. The drawbacks of low-threshold devices include the production of leakage currents and idle power dissipation. The switching noise in integrated circuits is circuit controlled by the accurate estimation of the harmonic contents of CMOS logic-switching waveforms. IC designers distribute the noise budget among different blocks of design, and this technique is helpful in the reduction of switching noise.

### BSIM Models

Many semiconductor manufacturing companies and academic institutes have developed their own transistor models and claim they are the best in the world. This situation makes it difficult to decide what the best transistor model is. Fortunately, UC Berkeley developed BSIM in 1990. BSIM transistor models accurately fit into the I–V characteristic curves of modern transistors. The BSIM 3 transistor model contains more than 200 parameters that affect the simulation results and are an essential part of the device physics of modern transistors. These parameters model the second-order effects of modern transistors. BSIM transistor models are reliable and accurate; however, they do not appropriately model the leakage currents of modern transistors. BSIM 4 is an extension of the BSIM 3 model, and BSIM 5 and BSIM 6 are further extensions. BSIM transistor models provide detailed information about all second-order effects such as drain-induced barrier lowering (DIBL), mobility degradation, short-channel effects, velocity saturation, etc. Modern BSIM transistor models have multiple gate capacitance models such as resistance models and diffusion capacitance. The temperature dependence of diode junction capacitance is also included in modern BSIM transistor models. Modern BSIM transistor models include parameter checking in the package to avoid bad parameter values.

## 3. Switching Noise

Switching noise is a complex issue in the design of digital integrated circuits. Switching noise consists of three different parts: the first part is the circuit that generates noise, called the aggressor circuit. The second part is the circuit that couples the noise from the aggressor, called the victim circuit. The third part is the circuit that picks the noise from the victim circuit. The total noise, received at the victim circuit, is a combination of noise from many aggressor circuits. Circuit reduction techniques are important in noise generation. Reduced digital circuits generate less noise, and large digital circuits generate more noise, for which we require large computer and software resources to simulate.

To control noise generation in digital integrated circuits, the study of harmonic contents of the switching waveform spectrum is essential. The time-domain waveform of a signal determines its spectrum. According to the rule stated by Lee [11]: “the spectrum of a signal will decay as $1/f^{n}$, where n is the number of derivatives of the signal required to yield an impulse.” The existence of discontinuities in the model distorts the spectra, and hence, the discontinuities occur only in the simulation domain. To study the higher-order harmonics in the spectrum of digital circuits, we need to avoid discontinuities in the models; otherwise, the results will not be trustworthy.

Regardless of the shape of the input waveform, there will be higher-order harmonics in all output-switching waveforms generated by the digital circuits, as output waveforms approximately resemble square waves. Due to comparatively slow edges, sinusoid-shaped waves produce extra short-circuit power. Higher-order harmonics are also generated when the sinusoid-shaped waves are amplified and limited. Due to sharp edges, square-shaped waves create discontinuity in the output-switching waveform or its derivatives, which decreases the spectrum roll-off of the output-switching waveforms. The best candidate for input to digital circuits is a square-like wave (neither sinusoid-shaped nor perfect-square-shaped). Hence, we generated a square-like wave by using the mathematical function of arctan(sin). The arctan(sin) function produces a continuous waveform, as both the tangent and sine functions are continuous.

## 4. Tool Setup and Input Signal Selection

**Figure 1:**
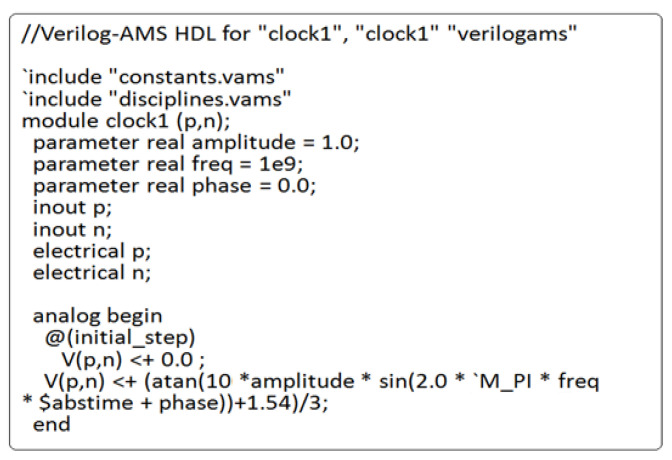
Verilog-A code for input signal generation.

By using the Verilog-A tool, we generated a square-like wave with the mathematical arctan(sin) function. Figure 1 shows Verilog-AMS HDL code for the generation of the input signal.

V = arctan (10 × sin(x))(1)

### 4.1. Simulations Using 65 nm Process Technology

**Figure 2:**
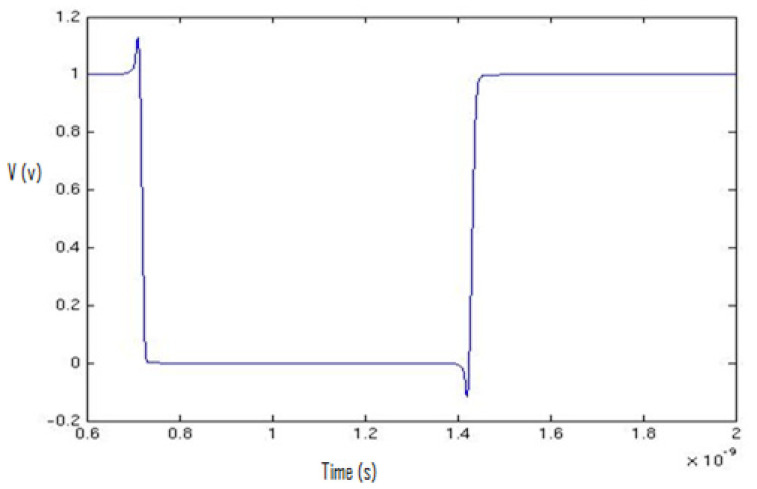
Inverter output plot (one cycle) in MATLAB.

For the tool set-up, we started the simulations of a simple inverter, and then we extended the idea of the simulations to complex flip-flop and latch structures. First, we plotted the waveforms using the Cadence Spectre Simulator and then transferred the equidistant data points to MATLAB, as shown in Figure 2. We used high accuracy settings in the Cadence Spectre to obtain 5000 equidistant data points in a specific cycle to obtain a clear view of data points in the switching spectrum. After that, we plotted the spectrum of the specific cycle that contained 5000 equidistant data points using MATLAB.

**Figure 3:**
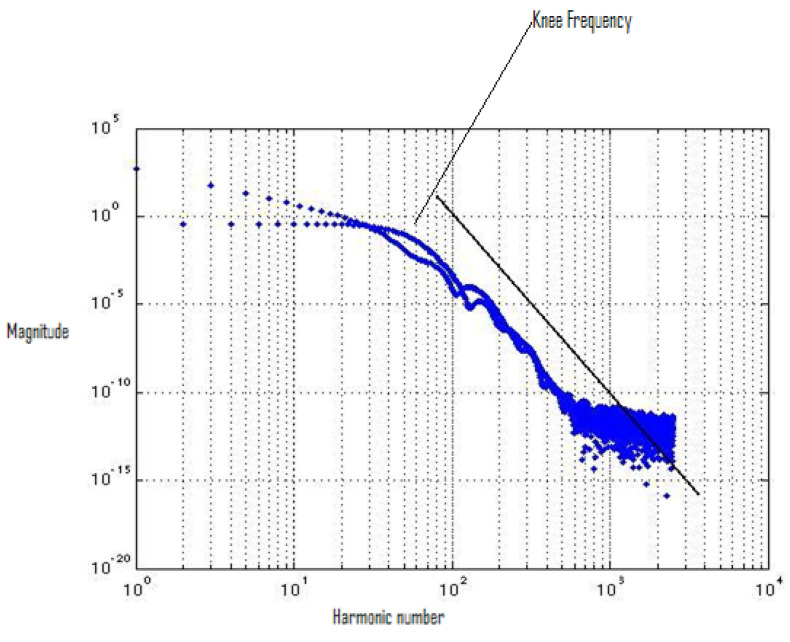
Spectrum plot of the inverter switching waveform.

By using FFT, we evaluated the spectrum of the output-switching waveform. Figure 3 describes the switching waveform of an inverter using a 65 nm process technology design kit. The 65 nm process design technology kit uses the BSIM 4 transistor model. Figure 3 shows the spectrum roll-off of 20 dB/decade before the knee frequency, and beyond the knee frequency, the spectrum roll-off is 100 dB/decade, which is much steeper. At knee frequency, the spectral amplitude is down by half (−6.8 dB) below the natural 20 dB/decade roll-off [11]. A 100 dB/decade spectrum roll-off means that there is no discontinuity in the first four derivatives of the provided input signal. We used the input signal of the square-like wave generated by the arctan(sin) wave to ensure that there was no discontinuity in the input signal. Discontinuity decreases the spectrum roll-off and generates higher-order harmonics. To avoid higher-order harmonics, a steeper roll-off of the switching spectrum is desirable, and to control noise in digital circuits, the correct estimation of harmonics is required [12,13,14,15,16,17].

### 4.2. Simulations Using 130 nm Process Technology

**Figure 4:**
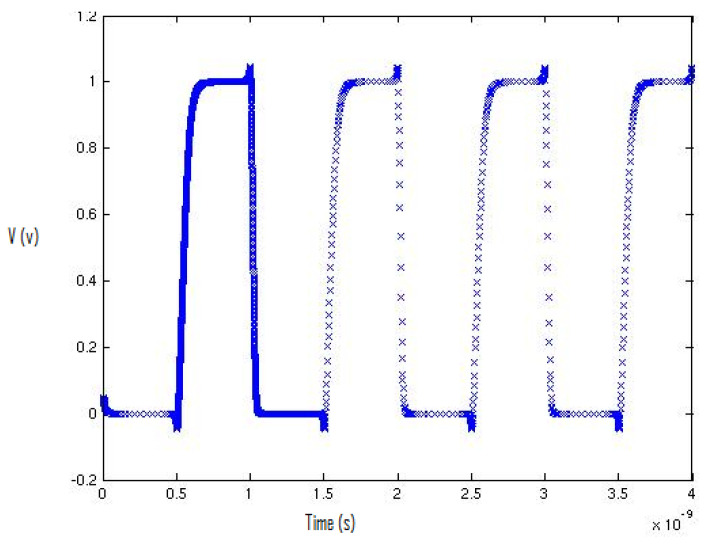
Output plot of the inverter in MATLAB: the first cycle of the waveform contains 5000 equidistant data points.

Figure 4 describes the inverter’s output-switching waveform using the 130 nm process. The first cycle of the waveform contains 5000 equidistant data points.

#### First Cycle Waveform:

**Figure 5:**
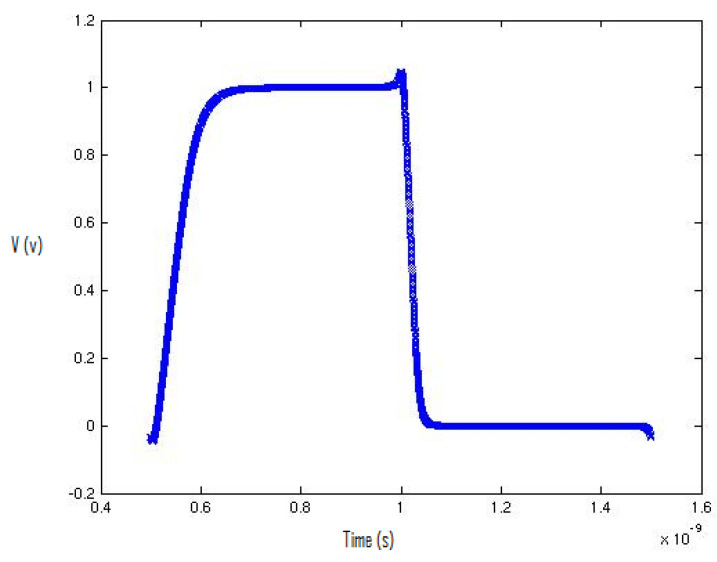
Inverter output plots (one cycle) in MATLAB.

Figure 5 shows the plot of the first cycle of the switching waveform of the inverter in MATLAB.

**Figure 6:**
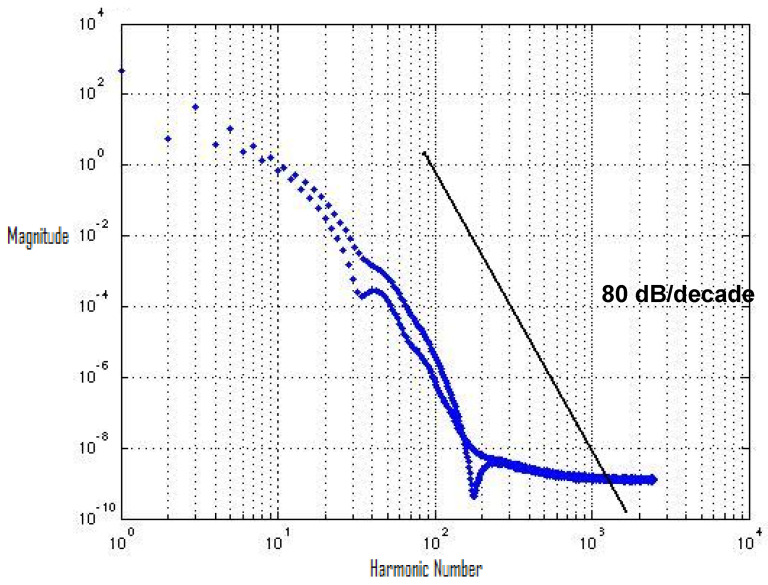
Spectrum plot of the inverter’s switching waveform.

Figure 6 shows the MATLAB spectrum plot of the first cycle of the switching waveform of the inverter. The spectrum roll-off is around 80 dB/decade by using the 130 nm process design kit.

### 4.3. Comparison

Table 1 compares the simulation results of 65 and 130 nm process technologies in terms of the rise time and roll-off of the switching spectrum waveforms.

## 5. Flip-Flop and Latch Topologies

For our analysis, we selected a set of latches and flip-flops that were designed to construct high-performance and low-power microprocessors [14,15,18]. To meet our design goals, we applied:

- A pulsed design;
- Smallest as possible direct path;
- Smallest as possible node swing;
- Smallest as possible clock load;
- Low-power feedback and optimized master–slave stages.

To meet our design objectives, we did not want the setup time of the design to be positive, clock slope and clock skew sensitive, dynamic and floating nodes and master latch as dynamics.

**Figure 7:**
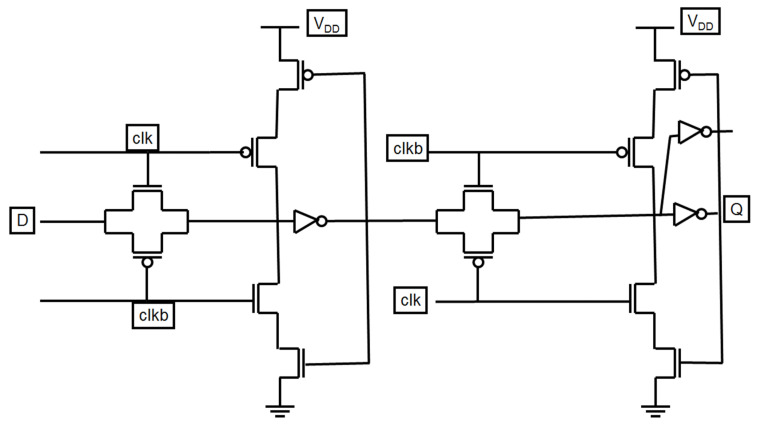
Schematic of the PowerPC 603 master–slave latch.

The PowerPC 603 master–slave latch is a fast classical structure, as shown in Figure 7. The PowerPC 603 master–slave latch has a short direct path and low-power feedback, which makes it ideal for use in high-performance microprocessors. However, the clock load of the PowerPC 603 master–slave structure is large, which increases the total power in-chip dissipation.

**Figure 8:**
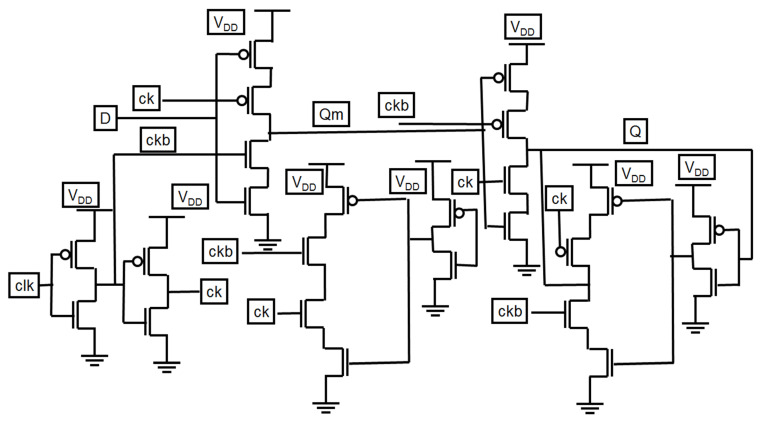
Schematic of the modified C²MOS latch.

Figure 8 presents a modified C^2^MOS latch structure. The modification in the dynamic C^2^MOS master–slave latch [14,15,18,19,20] results in better low-power characteristics and a small clock load, obtained by the low-power feedback and local clock buffering, assuring fully static operation. The PowerPC 603 master–slave latch is faster than the modified C^2^MOS master–slave latch. The PowerPC master–slave latch uses complementary pass-gate transistors, which results in a faster pull-up and increase in the sensitivity to race through one gate delay period in which both phases overlap.

**Figure 9:**
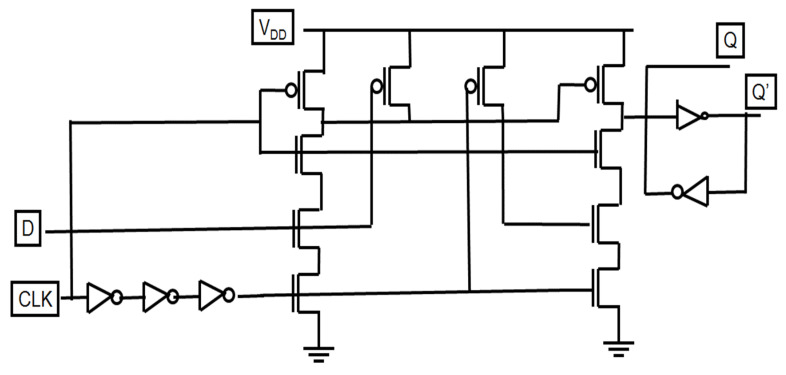
Schematic of the hybrid-latch flip-flop (HLFF).

Figure 9 presents a hybrid-latch flip-flop (HLFF) structure. Hybrid-latch flip-flop (HLFF) is one of the fastest structures discussed in this paper. The power delay product (PDP) of HLFF is very small. One of the main advantages of HLFF is its robustness to clock skew, and one of the disadvantages of HLFF is its positive hold time. Due to the single output design, the power dissipation of HLFF is almost similar to static logic circuits [14,15,21,22,23,24].

Other flip-flop and latch structures presented in this article include semi-dynamic flip-flop (SDFF), K6 edge-triggered latch (K6-ETL), StrongArm110, precharged sense-amplifier stage SA-F/F, static single-transistor-clocked flip-flop (SSTC) and dynamic single-transistor-clocked flip-flop (DSTC) [25,26]. SDFF is the fastest among all the structures presented in this article. The K6 edge-triggered latch is a differential, dynamic, self-resetting and hybrid structure. The K6 edge-triggered-latch is a fast structure, but it has a drawback, i.e., very high power consumption independent of the data pattern. Monotonous transition at the outputs of K6-ETL, SA-F/F and StrongArm110 FF is an important feature, which drives the fast domino logic. Due to the capacitive coupling effect, DSTC and SSTC suffer from voltage drop at the output.

## 6. Simulation and Results

We performed optimization. The first step is the identification of the critical path. Using the Brent–Powell optimization algorithm embedded in the Cadence Virtuoso ADE GXL* environment, the widths of the transistors were optimized on the critical path within the specified range (0.065 to 0.90 µm for 65 nm) and (0.15 to 2.06 µm for 130 nm) for minimum total power and Clk-Q delay as shown in Table 2.

Transistor widths that connected in the feedback paths were kept minimum.

Simulation results using 65 and 130 nm process nodes are shown below in Table 3, Table 4, Table 5, Table 6, Table 7 and Table 8.

**Figure 10:**
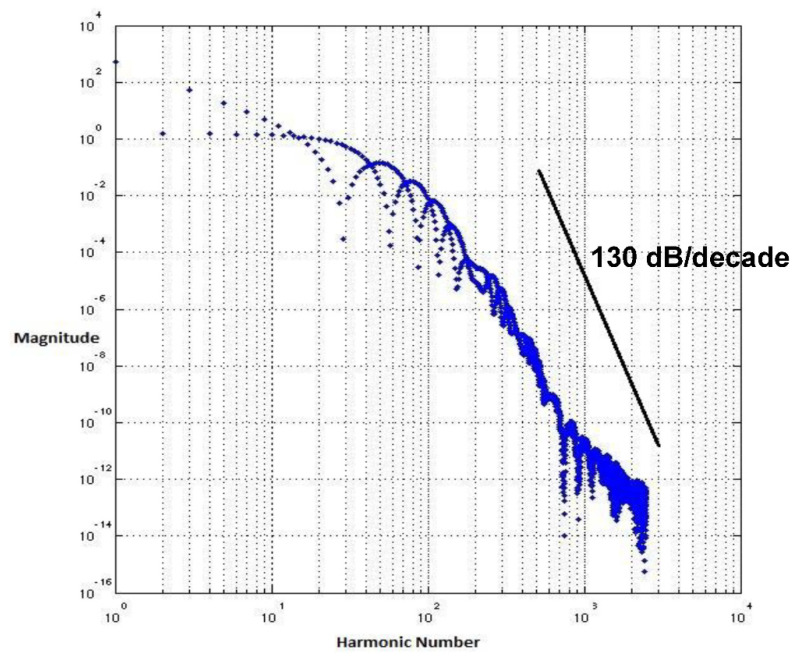
Spectrum roll-off of HLFF using the 65 nm process.

**Figure 11:**
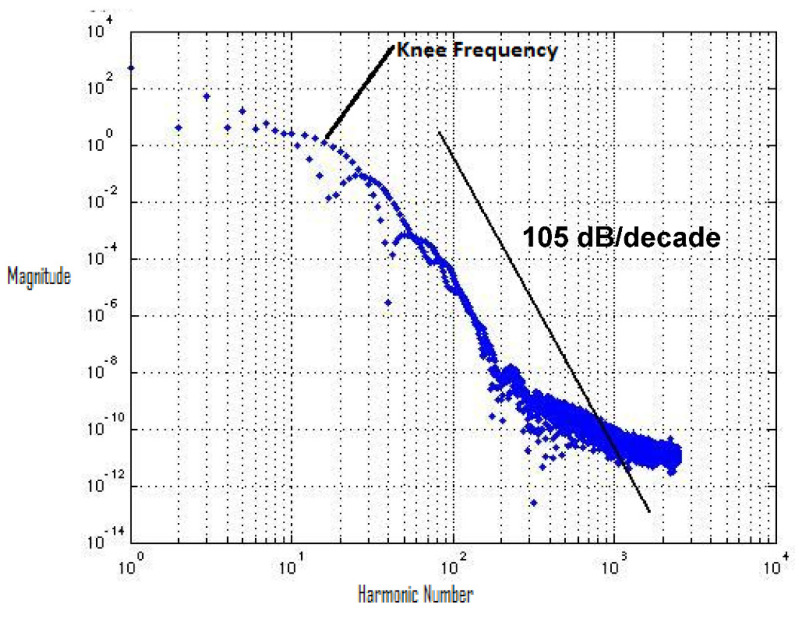
Spectrum roll-off of HLFF using the 130 nm process.

**Figure 12:**
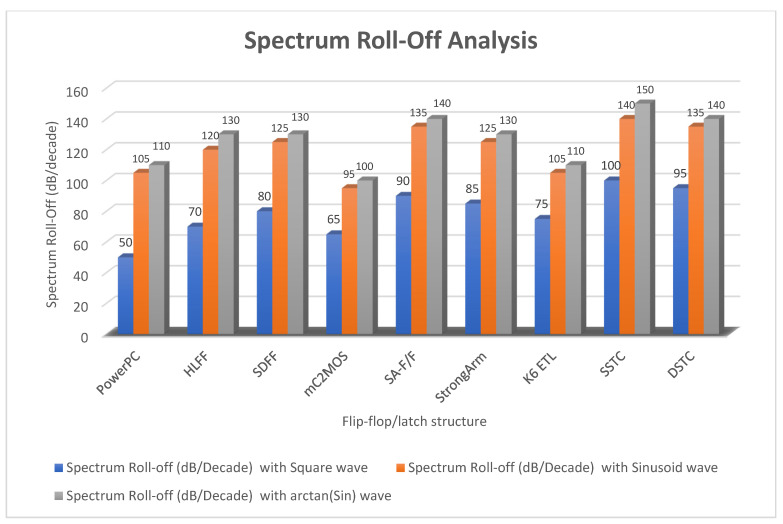
Spectrum roll-off analysis using 65 nm process technology and various types of input signals.

**Figure 13:**
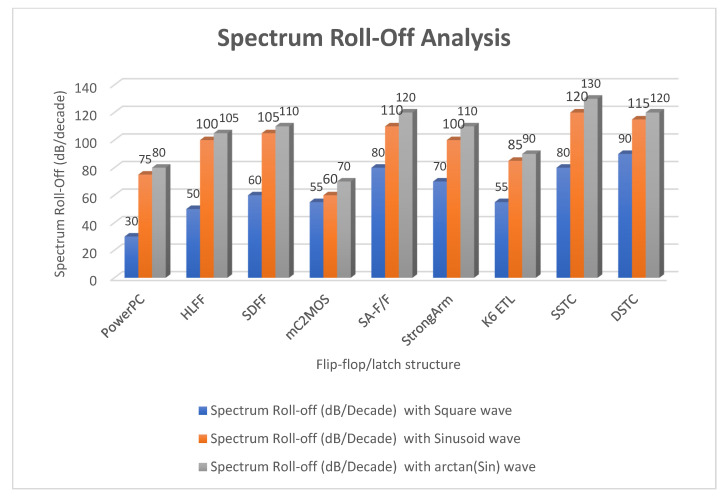
Spectrum roll-off analysis using 130 nm process technology and various types of input signals.

**Figure 14:**
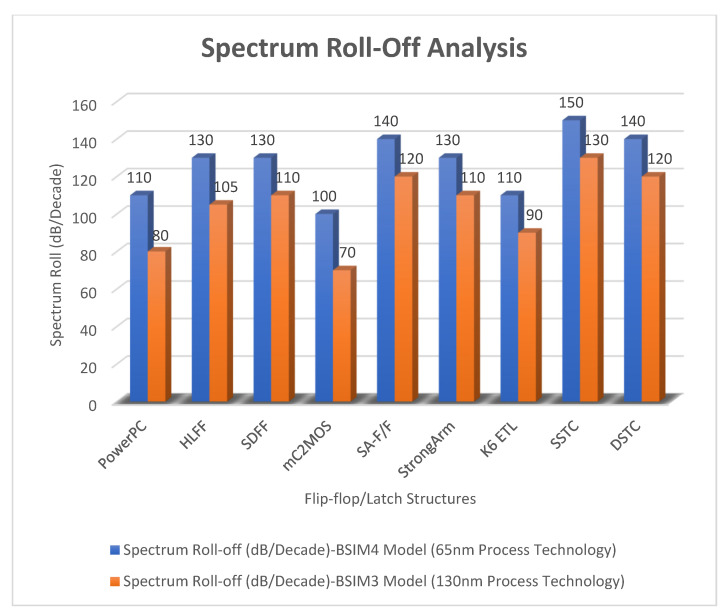
Spectrum roll-off analysis using 65 and 130 nm process design kits and arctan(sin) as the input signal.

There is a significant improvement in the spectrum roll-off with BSIM4 models using the 65 nm process. Figure 10 presents the spectrum of switching waveform of the hybrid-latch flip-flop (HLFF) using 65 nm process technology. The spectrum roll-off of HLFF is about 20 dB/decade before the knee frequency and after the knee frequency. The spectrum roll-off of HLFF is around 130 dB/decade, which is much faster than 20 dB/decade. At knee frequency, the spectral amplitude is down by half (−6.8 dB) below the natural 20 dB/decade roll-off [27]. The spectrum roll-off of 130 dB/decade shows that there is no discontinuity in the first five derivatives of the output signal. Figure 11 presents the spectrum of switching waveform of the hybrid-latch flip-flop (HLFF) using 130 nm process technology. The HLFF spectrum roll-off using 130 nm process technology is 105 dB/decade (105 dB/decade spectrum roll-off indicates that there is no discontinuity in the first four derivatives of the output signal of HLFF). Figure 12 and Figure 13 present the spectrum roll-off analysis using 65 and 130 nm process technologies with different types of input signals. Figure 14 presents the spectrum roll-off analysis using 65 and 130 nm process design kits and arctan(sin) as the input signal. To avoid higher-order harmonics, we want the spectrum roll-off of the switching waveform to be as steep as possible.

## 7. Power Dissipation Analysis

Power dissipation is a major problem in digital integrated circuits. It decreases the performance and reliability of digital integrated circuits; hence, low-power designs are a major challenge for digital designers.

**Figure 15:**
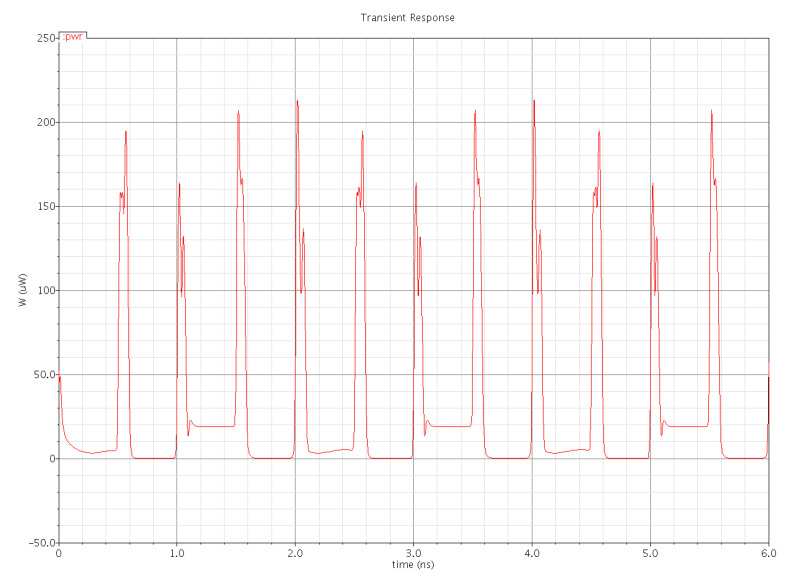
Total power dissipation in the PowerPC 603 master–slave latch.

Power dissipation in a digital integrated circuit is mostly dependent on its structure and statistics of the data signal applied. Figure 15 shows the plot of the total power dissipation of the PowerPC 603 master–slave latch. The total power dissipation is the sum of three power dissipations, i.e., internal power dissipation of the latch, local clock power dissipation and local data power dissipation. In Figure 15, the pulse-train-like appearance of the plot is due to switching activity. When no switching happens, the signal remains unchanged, and the dynamic power is zero, but rapidly changing signals provoke plenty of switching, and therefore, power dissipation occurs. We applied a voltage of 1 V for this simulation. The average power dissipation of the plot is 25.08 µW. In some intervals, the power dissipation waveform does not go back to zero because either the static current flow or the capacitors remain partially charged during the interval.

**Figure 16:**
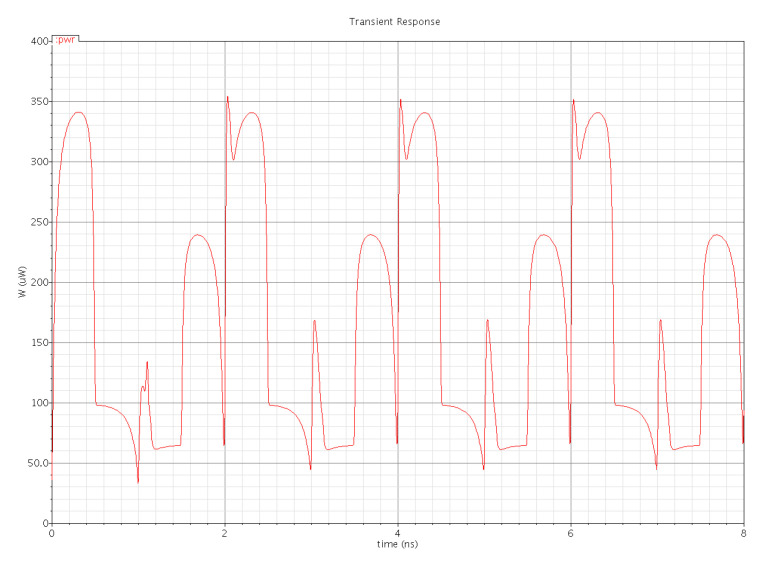
Total power dissipation in the modified C²MOS latch.

**Figure 17:**
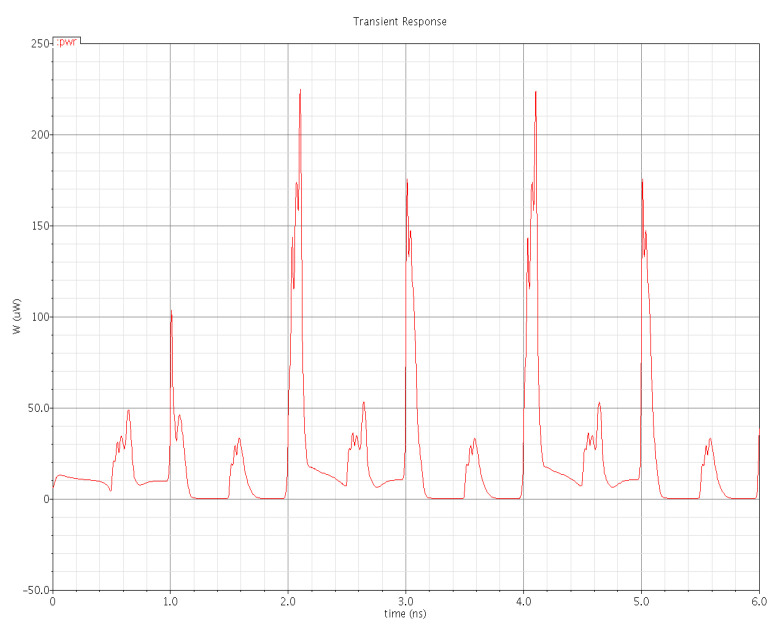
Total power dissipation in the hybrid-latch flip-flop (HLFF).

**Figure 18:**
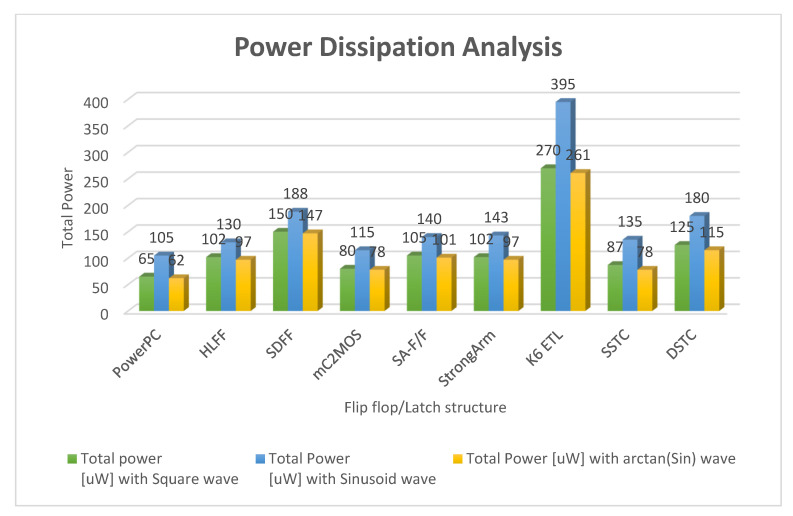
Total power dissipation analysis using 65 nm process technology and various types of input signals.

**Figure 19:**
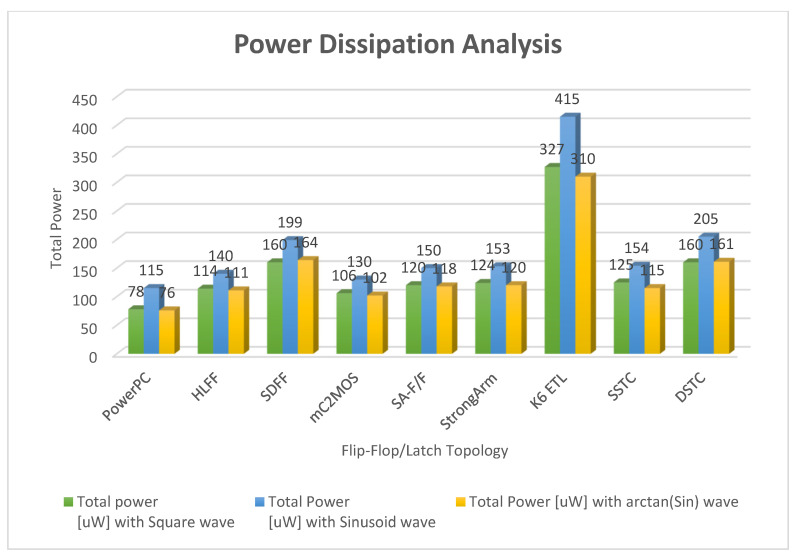
Total power dissipation analysis using 130 nm process technology and various types of input signals.

**Figure 20:**
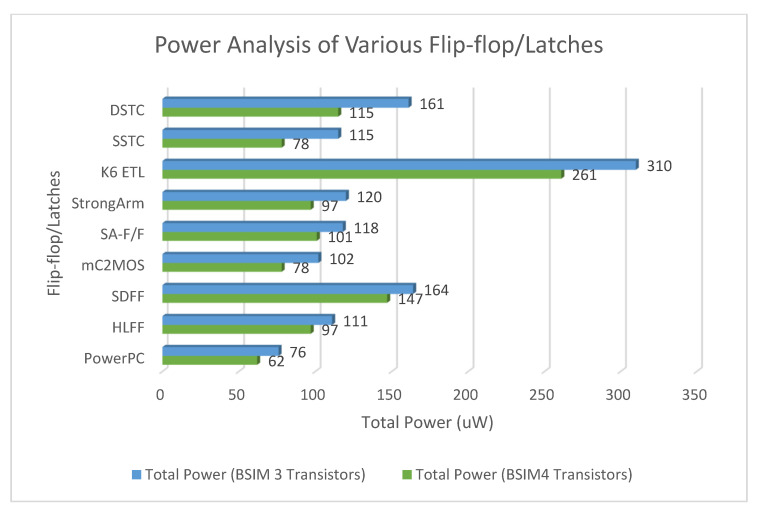
Power Analysis of various flip-flop/latch topologies using 65 and 130 nm process technologies.

Figure 16 and Figure 17 show the waveforms of power dissipations in the modified C²MOS latch and hybrid-latch flip-flop (HLFF). Figure 18 and Figure 19 show the total power dissipation analysis using 65 and 130 nm process technologies and various types of input signals. Figure 20 shows the power analysis of various flip-flop and latch topologies using 65 and 130 nm process technologies and arctan(sin) as the input signal.

Transistor model quality and input signal selection play an important role in the accurate estimation of harmonic contents of switching waveforms. We used a square-shaped signal as an input to our simulations (neither a sinusoid nor a perfect square wave). A perfect square wave causes discontinuity in the spectrum due to abrupt change, and a sinusoid-shaped wave has slower edges, which produce extra short-circuit power. The discontinuity reduces the spectrum roll-off of switching waveform, and the higher-order harmonics generate noise contents [28,29,30,31,32,33,34,35].

**Figure 21:**
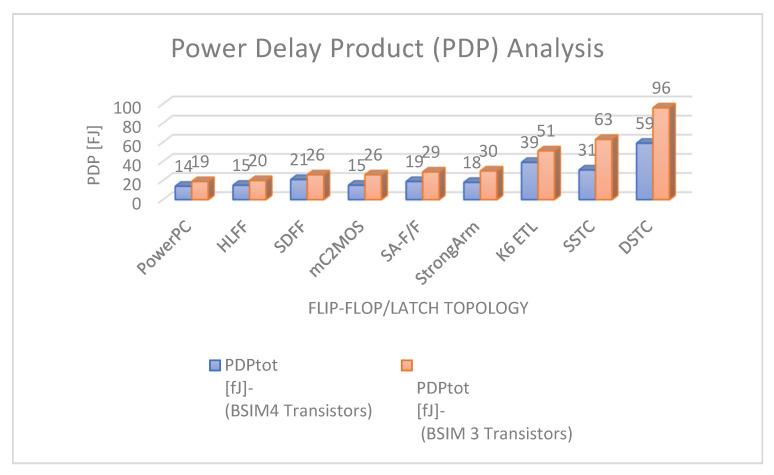
Power delay product (PDP).

In this paper, we presented simulation results of the spectrum roll-off (dB/decade) of nine different flip-flops using BSIM3 and BSIM4 transistor models in two different CMOS process technologies (130 and 65 nm). To control digital noise in digital integrated circuits, the exact estimation of switching harmonics is essential. The total power dissipation in the flip-flop/latch structures is the sum of three power dissipations, i.e., internal power dissipation, local clock and local data power dissipation. The PowerPC 603 master–slave latch dissipates small power compared to the other flip-flop and latch structures, and it has a switching spectrum roll-off of 115 dB/decade for the 65 nm process design kit. SDFF is the fastest structure among all topologies presented with a delay of 140 ps, and DTSC is the slowest structure, with a delay of 510 ps in the 65 nm process. SSTC provides a steeper roll-off of 150 dB/decade in the 65 nm process. The spectrum roll-off of 150 dB/decade shows that there is no discontinuity in the first six derivatives of the output signal. Figure 18 and Figure 19 present the total power dissipation analysis using 65 and 130 nm process technologies, respectively, using various types of input signals. Figure 20 presents the power analysis using various flip-flop/latch topologies. Figure 21 presents the power delay product (PDP) of all nine flip-flop and latch topologies discussed in this paper.

## 8. Conclusions

The analysis presented in this research article can help the designer to design a highly integrated noise-controlled digital CMOS circuit for use in future-generation micro- and nanoelectromechanical systems. An accurate estimate of higher-order harmonic contents is essential in the design and development of highly integrated wireless communication systems. Harmonic content estimations help in designing tapered buffer chains, but the design quality influenced by the device model has discontinuities. Based on the findings discussed in this research article, we believe that the selection of input signal and transistor model quality is critically important for the accuracy of noise estimates. We used Cadence Spectre and MATLAB to analyze the spectrum of different flip-flops and latch topologies. They showed steep spectrum roll-off after knee frequency. As a concrete example, various kinds of latches and flip-flops, which have the fastest switching capability, were evaluated by using BIM3v3 and BSIM4 models. Higher-order harmonics generate noise contents, and discontinuity decreases the switching waveform spectrum roll-off. Using a quality transistor model and slow-changing input signal aids in reducing the discontinuity and hence gives us control of higher-order harmonics. The flip-flops and latches designed in the 65 nm process using the BSIM4 model are evident of reduced discontinuity, which further means the least impact of noise interference.

## Figures and Tables

**Table 1:** Comparison of the simulation results.

S/No.	Process Technology	Switching Spectrum Roll-Off	Rise Time
1	65 nm	100 dB/decade	0.3 ns
2	130 nm	80 dB/decade	0.7 ns

**Table 2:** CMOS simulation parameters.

Parameters	0.13 µm CMOS Technology	0.065 µm CMOS Technology
Minimum Gate Width	0.15 µm	0.065 µm
Maximum Gate Width	2.06 µm	0.90 µm
MOSFET Model	BSIM 3v3	BSIM4
Nominal Conditions	V_DD_ = 1 V Temperature = 27 °C	V_DD_ = 1 V Temperature = 27 °C
Clock Frequency	1 GHz	1 GHz
Data Clock Frequency	500 MHz	500 MHz

**Table 3:** Simulation results using the BSIM4 transistor model (65 nm process technology).

Parameters	PowerPC 603 Master–Slave Latch	mC^2^ MOS Latch	Hybrid-Latch FF
Set-up Time in ns (Minimum Value)	0.05	0.06	−0.02
Hold Time in ns (Minimum value)	0.04	0.05	14.00
Clk-Q Delay in ns	0.04	0.05	0.04
Peak Value of Power Dissipation (µW)	97.78	195.22	168.20
Average Value of Power Dissipation (µW)	11.67	110.80	13.20
Spectrum Roll-Off (dB/decade)	110	100	130

**Table 4:** Simulation results using the BSIM4 transistor model.

Flip-Flop/Latch Topology	Number of Transistors	Internal Power (uW)	Clock Power (uW)	Data Power (uW)	Total Power (uW)	Delay (ps)	PDP_tot_ (fJ)	Spectrum Roll-Off (dB/Decade)
PowerPC	16	30	28	4	62	220	14	110
HLFF	20	86	8	3	97	150	15	130
SDFF	23	132	13	2	147	140	21	130
mC^2^MOS	24	66	7	5	78	198	15	100
SA-F/F	19	89	10	2	101	186	19	140
StrongArm	20	85	10	2	97	190	18	130
K6 ETL	37	250	7	4	261	150	39	110
SSTC	16	65	10	3	78	400	31	150
DSTC	10	102	10	3	115	510	59	140

**Table 5:** Comparison of simulation results using the BSIM4 transistor model.

Flip-Flop/Latch Topology	P_tot_ (uW) with Square Wave	P_tot_ (uW) with Sinusoid Wave	P_tot_ (uW) with arctan(sin) Wave	Spectrum Roll-Off (dB/Decade) with Square Wave	Spectrum Roll-Off (dB/Decade) with Sinusoid Wave	Spectrum Roll-Off (dB/Decade) with arctan(sin) Wave
PowerPC	65	105	62	50	105	110
HLFF	102	130	97	70	120	130
SDFF	150	188	147	80	125	130
mC^2^MOS	80	115	78	65	95	100
SA-F/F	105	140	101	90	135	140
StrongArm	102	143	97	85	125	130
K6 ETL	270	395	261	75	105	110
SSTC	87	135	78	100	140	150
DSTC	125	180	115	95	135	140

**Table 6:** Simulations results using the BSIM3 transistor model (130 nm process technology).

Parameters	PowerPC 603 Master–Slave Latch	mC^2^ MOS Latch	Hybrid-Latch FF
Set-up Time in ns (Minimum Value)	0.11	0.88	−0.070
Hold Time in ns (Minimum value)	0.13	0.11	0.20
Clk-Q Delay in ns	0.06	0.06	0.11
Peak Value of Power Dissipation (µW)	210	352	222
Average Value of Power Dissipation (µW)	25.08	181.6	21.18
Spectrum Roll-Off (dB/decade)	80	70	105

**Table 7:** Simulation results of the BSIM3 transistor model.

Flip-Flop/Latch Topology	Number of Transistors	Internal Power (uW)	Clock Power (uW)	Data Power (uW)	Total Power (uW)	Delay (ps)	PDP_tot_ (fJ)	Spectrum Roll-Off (dB/Decade)
PowerPC	16	40	32	4	76	244	19	80
HLFF	20	98	10	3	111	178	20	105
SDFF	23	144	18	2	164	158	26	110
mC^2^MOS	24	88	9	5	102	254	26	70
SA-F/F	19	104	12	2	118	244	29	120
StrongArm	20	106	12	2	120	246	30	110
K6 ETL	37	296	10	4	310	166	51	90
SSTC	16	98	14	3	115	550	63	130
DSTC	10	144	14	3	161	595	96	120

**Table 8:** Comparison of simulation results using the BSIM3 transistor model.

Flip-Flop/Latch Topology	P_tot_ (uW) with Square Wave	P_tot_ (uW) with Sinusoid Wave	P_tot_ (uW) with arctan(sin) Wave	Spectrum Roll-Off (dB/Decade) with Square Wave	Spectrum Roll-Off (Db/Decade) With Sinusoid Wave	Spectrum Roll-Off (dB/Decade) with arctan(sin) Wave
PowerPC	78	115	76	30	75	80
HLFF	114	140	111	50	100	105
SDFF	160	199	164	60	105	110
mC^2^MOS	106	130	102	55	60	70
SA-F/F	120	150	118	80	110	120
StrongArm	124	153	120	70	100	110
K6 ETL	327	415	310	55	85	90
SSTC	125	154	115	80	120	130
DSTC	160	205	161	90	115	120
